# Treatment Patterns and Survival in Locally Advanced or Metastatic Biliary Tract Cancer Using SEER Medicare Data

**DOI:** 10.1016/j.gastha.2023.01.009

**Published:** 2023-01-21

**Authors:** Mark D. Danese, Kabir Mody, Ramya Thota, Stacie C. Lindsey, Melinda Bachini, Reham Abdel-Wahab, François Audhuy, Jennifer Duryea, Sarah Bobiak

**Affiliations:** 1 Outcomes Insights Inc, Agoura Hills, California; 2 Division of Hematology/Oncology, Mayo Clinic, Jacksonville, Florida; 3 Intermountain Medical Oncology, Salt Lake City, Utah; 4 Cholangiocarcinoma Foundation, Herriman, Utah; 5 Clinical Oncology Department, Assiut University, Assiut, Egypt; 6 EMD Serono Research & Development Institute, Inc. (an affiliate of Merck KGaA), Billerica, Massachusetts

**Keywords:** Patient outcomes, Cholangiocarcinoma, Gallbladder cancer

## Abstract

**Background and Aims:**

Biliary tract cancer (BTC) is a rare, lethal, heterogeneous group of cancers often diagnosed at an advanced stage. While gemcitabine plus cisplatin is the standard of care for first-line treatment of locally advanced or metastatic BTC, no globally accepted standard of care currently exists for second-line treatment of BTC following chemotherapy. However, the treatment landscape is evolving with approvals for therapies targeting actionable mutations. This study aimed to characterize treatment patterns and survival in patients with locally advanced or metastatic BTC.

**Methods:**

Patients with advanced or metastatic BTC in the Surveillance, Epidemiology, and End Results Medicare database between 2010 and 2015 (N = 2063) were included; patients with nonprimary BTC were excluded. Patient and clinical characteristics, line and type of therapy, and overall survival of patients were analyzed.

**Results:**

Only 45.5% (n = 938) of patients initiated systemic therapy within 90 days of diagnosis. The most common event following diagnosis was initiation of first-line therapy, and the most common event following first-line treatment was death. Median survival ranged from 5.0 months for patients receiving second-line fluoropyrimidine to 9.7 months for patients receiving second-line gemcitabine. Duration of therapy ranged from 0.7 months for patients receiving second-line fluoropyrimidine to 3.7 months for patients receiving first-line gemcitabine plus cisplatin therapy.

**Conclusion:**

Overall survival from diagnosis was poor and influenced by age, sex, stage, mobility limitations, comorbidity burden, poverty, and previous cancer. Treatment patterns varied for patients who progressed following first-line therapy, as there was no consensus second-line treatment for locally advanced or metastatic BTC without clinically targetable mutations.

## Introduction

Biliary tract cancer (BTC) is a rare, highly heterogeneous, and lethal group of cancers that includes 4 subtypes: intrahepatic cholangiocarcinoma, extrahepatic cholangiocarcinoma (sometimes further divided into perihilar and distal cholangiocarcinomas), gallbladder cancer, and ampullary cancer.^1^^,^^2^ Gallbladder cancer is the most common BTC, followed by extrahepatic cholangiocarcinoma, intrahepatic cholangiocarcinoma, and ampullary cancer, although the assessment of cholangiocarcinoma incidence is complicated by historical indexing of perihilar cholangiocarcinoma cases (a component of extrahepatic cholangiocarcinoma) as intrahepatic cholangiocarcinoma.^3^^,^^4^ Gallbladder cancer is also the most lethal subtype of BTC. Ampullary cancer is sometimes included in BTC studies due to its pancreatobiliary origin but is sometimes treated differently than other biliary cancers. Globally in 2017, approximately 211,000 new cases and 174,000 estimated deaths were due to BTC.^5^ The vast majority of patients with BTC are diagnosed at an advanced stage and present with nonspecific symptoms, which may result in misdiagnosis or delayed diagnosis that can lead to worse prognosis.^6^, ^7^, ^8^

The recommended first-line standard of care for unresectable, locally advanced, or metastatic BTC is gemcitabine plus cisplatin, based on 2 randomized studies and a meta-analysis of both studies.^8^, ^9^, ^10^, ^11^ Currently, no globally accepted standard-of-care treatment exists for locally advanced or metastatic BTC for which standard chemotherapy has failed.^12^ While the second-line treatment landscape is evolving with recent approvals for targeted therapies, for the majority of patients, chemotherapy remains the only option. A meta-analysis of 25 studies of second-line chemotherapy in patients with BTC showed a low response rate (objective response rate [ORR] 7.7%), with a median overall survival of 7.2 months and a median progression-free survival of 3.2 months.^12^ Modified FOLFOX (leucovorin, fluorouracil, and oxaliplatin) is recommended by the National Comprehensive Cancer Network based on recent data from a phase 3 trial among UK patients with locally advanced or metastatic BTC (ORR 5%; median overall survival, 6.2 months).^8^^,^^13^

Although some patterns of care and outcomes have been reported for subtypes of BTC, additional work is needed to better understand population characteristics and patterns of care in the treatment of locally advanced or metastatic BTC. Previous Surveillance, Epidemiology, and End Results (SEER) studies have looked at racial, ethnic, and age disparities in both the incidence and mortality of intrahepatic cholangiocarcinoma.^14^^,^^15^ Survival outcomes have also been improved with chemotherapy in patients with intrahepatic cholangiocarcinoma and gallbladder cancer based on SEER data.^16^ The objective of this study is to characterize patient and clinical characteristics, comorbid conditions, and treatment patterns and to estimate overall survival in patients with locally advanced or metastatic BTC using SEER Medicare data from January 2010 to December 2015.

## Methods

### Study Design

This retrospective, observational cohort study used data from the National Cancer Institute’s (NCI) SEER cancer registry linked with Medicare enrollment and claims data and was conducted in patients diagnosed with locally advanced or metastatic biliary tract cancer, including gallbladder cancer, extrahepatic cholangiocarcinoma, and intrahepatic cholangiocarcinoma. Ampullary cancer is not included in the National Comprehensive Cancer Network treatment guidelines for hepatobiliary cancers. Due to the unique histopathologic types, the variability in treatment and the different treatment outcomes for ampullary cancer,^3^ it was not included in this analysis.

### Patient Population

Patients included in this study were in the SEER Medicare database and diagnosed with locally advanced or metastatic BTC between January 1, 2010, and December 31, 2015, with follow-up Medicare claims through December 31, 2016 (Supplementary Figure 1). BTC subtypes were identified using the International Classification of Disease for Oncology topography codes C22.1 (intrahepatic cholangiocarcinoma), C23.9 (gallbladder cancer), and C24.0 (extrahepatic cholangiocarcinoma). The patient’s index date was the first day of the month in which they were diagnosed with BTC.

Inclusion criteria for this analysis were that all patients were aged ≥ 66 years at cancer diagnosis, had at least 365 days of Medicare eligibility prior to their index date and Medicare Part A and Part B coverage, and had no health maintenance organization coverage for the 365 days prior to their index date. In addition, all patients had microscopically confirmed cancer, and BTC was the patient’s first primary cancer. Patients were excluded if they had stage III disease and received resection with curative intent, had nonprimary BTC, had an unknown American Joint Committee on Cancer stage, or were diagnosed at autopsy or through a death certificate.

### Definition of Lines of Therapy

Systemic therapy use was based on Medicare claims including both Healthcare Common Procedure Coding System codes and National Drug Codes. Systemic therapies for BTC were defined as any chemotherapy, targeted therapy, or immunotherapy agents used by ≥ 3 patients. Initiation of first-line systemic outpatient therapy was defined as having received ≥ 1 systemic anticancer agent. For the purposes of this analysis, patients who did not receive outpatient systemic therapy or who received inpatient treatment only or who initiated systemic therapy after receiving hospice care were classified as “untreated” from the perspective of whether they used outpatient systemic therapy. Initiation of second-line therapy was defined as complete cessation of all agents from previous line and initiation of a different systemic therapy for BTC within 60 days or gap of ≥ 60 days after the previous line and initiation of any systemic therapy for BTC (including previous therapy). Switching from one platinum agent to another (eg, cisplatin to carboplatin) was not considered to be initiation of a new line of therapy.

### Analyses

For patients who received a subsequent line of therapy, the end date of their previous therapy was known and defined as the last treatment date for that line. For patients who did not receive a subsequent line of therapy, the end date was not always known because of possible censoring and was defined using information from the 60-day window after the last known treatment date. If death or censoring occurred in the 60-day window, then this death or censoring date was taken as the end date. If no death or censoring occurred in the interval, the most recent treatment date was considered as the end date. For patients who received treatment on only one date, the end date was imputed to be 7 days after the start date.

Patients were followed until death, disenrollment in Medicare Part A and Part B coverage, secondary cancer diagnosis, or the end of observation (December 31, 2016). Cox proportional hazards models were used to estimate associations with survival for each treatment cohort. The NCI comorbidity index was calculated using the algorithms defined by the NCI.^17^ Initial therapy and therapy after completion of first-line treatment were evaluated using stacked time-to-event plots. These plots show the probability of the following mutually exclusive states over time: hospice entry, inpatient systemic therapy, first-line systemic therapy, second-line systemic therapy, or subsequent cancer. All statistical analyses were performed using R (version 3.6.3), and data extraction/table creation was performed using Jigsaw software. Due to NCI privacy requirements, subgroups with < 11 patients are not shown.^18^

## Results

This cohort study consisted of patients with advanced or metastatic BTC in the SEER database (N = 2063). The median age of the advanced or metastatic BTC cohort was 76.1 years. Of these patients, 41.5% were male and 81.5% were White; 43.0% had gallbladder cancer, 28.8% had intrahepatic cholangiocarcinoma, and 28.2% had extrahepatic cholangiocarcinoma; 35.8%, 28.0%, and 36.2% had an NCI comorbidity category of 0, 1, and 2+, respectively. In the cohort, 25% had gallstones and 45.0% had either mobility limitations or a Charlson Comorbidity Index of 2 or more. Furthermore, 54.5% (n = 1125) received no outpatient systemic therapy, 31.7% (n = 654) received at least 1 line of systemic therapy, and 13.8% (n = 284) received at least 2 lines of systemic therapy (Table 1). Of the 1125 patients who were classified as not receiving initial outpatient systemic therapy, 61 (5%) received inpatient systemic therapy, 187 (17%) received hospice therapy, and 877 (78%) received neither. With a mean follow-up of 8 months, 93.6% of patients died during observation.

**Table 1:** Baseline Patient Characteristics

Characteristic	All patients at diagnosis (n = 2063)	No systemic therapy (n = 1125)	First-line (n = 938)	Second-line (n = 284)
Median age (SD), years	76.1 (6.9)	77.7 (7.3)	74.1 (5.7)	73.0 (5.1)
Sex, n (%)				
Female	1207 (58.5)	683 (60.7)	524 (55.9)	172 (60.6)
Male	856 (41.5)	442 (39.3)	414 (44.1)	112 (39.4)
Race, n (%)				
White	1682 (81.5)	906 (80.5)	776 (82.7)	242 (85.2)
Black	191 (9.3)	114 (10.1)	77 (8.2)	21 (7.4)
Asian	167 (8.1)	NR^a^	NR^a^	NR^a^
Other	23 (1.1)	NR^a^	NR^a^	NR^a^
AJCC stage, n (%)				
III	186 (9.0)	118 (10.5)	68 (7.2)	24 (8.5)
IV	1877 (91.0)	1007 (89.5)	870 (92.8)	260 (91.5)
Tumor site, n (%)				
EHCC	582 (28.2)	326 (29.0)	256 (27.3)	80 (28.2)
GBC	887 (43.0)	527 (46.8)	360 (38.4)	96 (33.8)
IHCC	594 (28.8)	272 (24.2)	322 (34.3)	108 (38.0)
NCI comorbidity index, n (%)^b^				
NCI category 0	739 (35.8)	358 (31.8)	381 (40.6)	131 (46.1)
NCI category 1	577 (28.0)	289 (25.7)	288 (30.7)	96 (33.8)
NCI category 2+	747 (36.2)	478 (42.5)	269 (28.7)	57 (20.1)
NCI index, mean (SD)	2.0 (2.2)	2.3 (2.4)	1.6 (2.0)	1.3 (1.8)
Comorbidity or mobility limitations, n (%)				
Any mobility limitation	505 (24.5)	353 (31.4)	152 (16.2)	42 (14.8)
Mobility limitation or Charlson Comorbidity Index 2+	928 (45.0)	586 (52.1)	342 (36.5)	85 (29.9)
Gallstones	516 (25.0)	299 (26.6)	217 (23.1)	61 (21.5)
Congestive heart failure	293 (14.2)	194 (17.2)	99 (10.6)	20 (7.0)
Renal disease	276 (13.4)	189 (16.8)	87 (9.3)	16 (5.6)
Liver disease	81 (3.9)	43 (3.8)	38 (4.1)	NR^a^
Cholangitis	48 (2.3)	29 (2.6)	19 (2.0)	NR^a^
Cholecystitis	169 (8.2)	97 (8.6)	72 (7.7)	24 (8.5)
Hearing loss	318 (15.4)	179 (15.9)	139 (14.8)	41 (14.4)
Peripheral neuropathy	12 (0.6)	NR^a^	NR^a^	0

AJCC, American Joint Committee on Cancer; EHCC, extrahepatic cholangiocarcinoma; GBC, gallbladder cancer; IHCC, intrahepatic cholangiocarcinoma; NCI, National Cancer Institute; NR, not reported.

a NCI privacy requirements dictate that populations < 11 cannot be reported.

b The NCI comorbidity index is a weighted count of comorbid conditions based on algorithms defined by NCI; higher scores indicate a higher risk of death due to comorbid conditions other than cancer, with a score of 0 indicating the presence of none of the 16 comorbid conditions.

**Figure 1:**
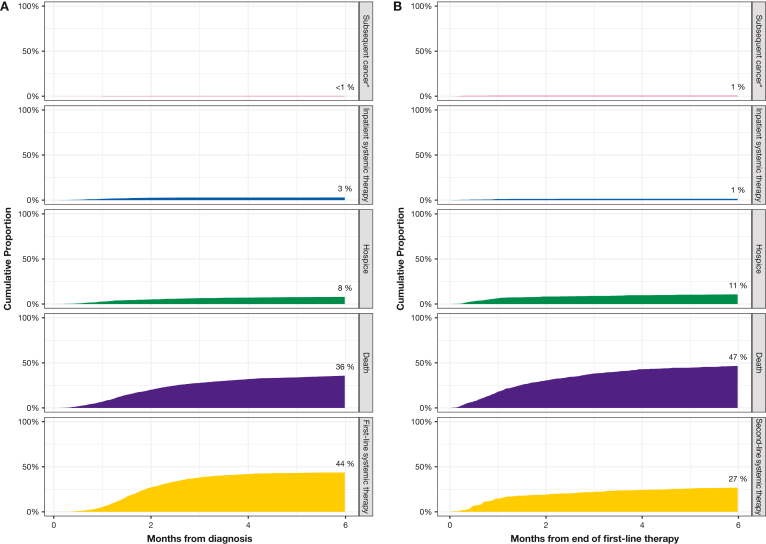
Time to first events after diagnosis (A) and after end of first-line therapy (B). ∗Subsequent cancer does not refer to metastasis, only an additional cancer diagnosis.

The most common milestone events within 6 months after diagnosis were first-line systemic therapy (44%) and death (36%), while hospice (8%), inpatient systemic therapy (3%), and subsequent cancer (< 1%) were less common (Figure 1A). Approximately 45% of patients had died or were in hospice care within 3–4 months of diagnosis. The most common milestone events within 6 months after the end of first-line systemic therapy were second-line systemic therapy (27%) and death (47%), while hospice (11%), inpatient systemic therapy (1%), and subsequent cancer (1%) were less common (Figure 1B). Overall, 18% of patients (n = 371) entered hospice during the study period.

**Figure 2:**
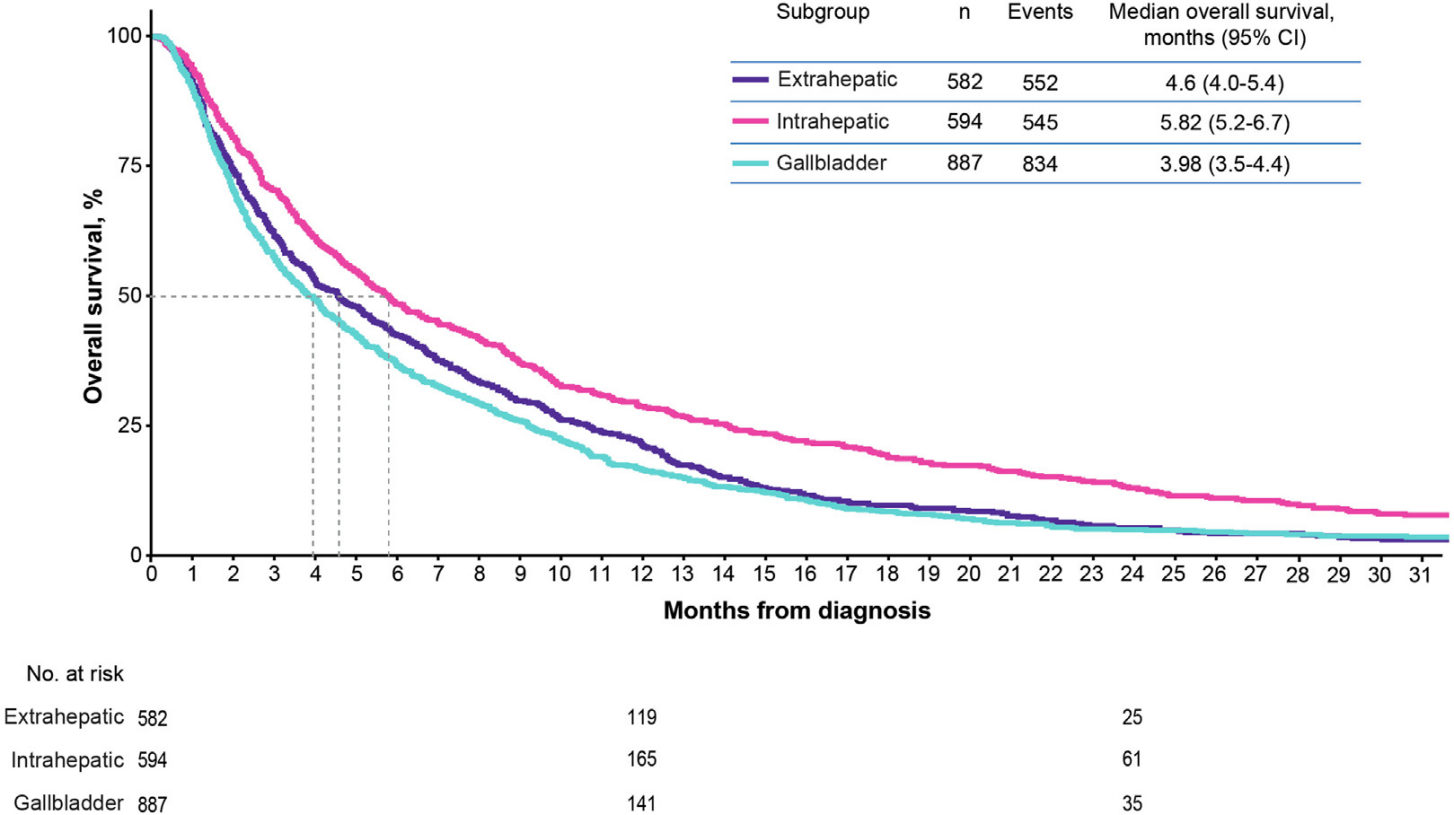
Overall survival from diagnosis by site of disease. CI, confidence interval.

**Figure 3:**
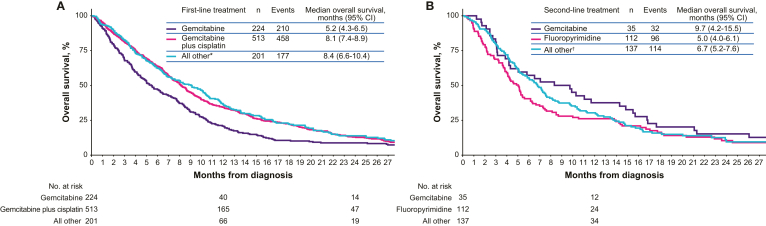
Overall survival for patients with biliary tract cancer receiving first-line (A) and second-line therapy (B). NCI, National Cancer Institute. ∗All other first-line treatment included fluoropyrimidine monotherapy (n = 93), fluoropyrimidine + platinum (n = 28), and fluoropyrimidine + gemcitabine (n = 23). The remaining therapies received by patients (n = 57) are censored per NCI privacy requirements (n > 11). ^†^All other second-line treatment included fluoropyrimidine + platinum (n = 27), gemcitabine + platinum (n = 26), fluoropyrimidine + irinotecan (n = 25). The remaining therapies received by patients (n = 59) are censored per NCI privacy requirements (n > 11).

The median overall survival for intrahepatic cholangiocarcinoma was the highest (5.8 months; 95% confidence interval [CI], 5.2–6.7 months), followed by extrahepatic cholangiocarcinoma (4.6 months; 95% CI, 4.0–5.4 months) and gallbladder cancer (4.0 months; 95% CI, 3.5–4.4 months) (Figure 2). Based on a Cox proportional hazards model, patients with intrahepatic cholangiocarcinoma vs extrahepatic cholangiocarcinoma had significantly improved survival, and patients with extrahepatic cholangiocarcinoma vs gallbladder cancer had improved survival (Supplementary Table 1). Other factors that were associated with worsened survival included older age, male sex, any mobility limitation, > 30% below poverty in census tract, and NCI comorbidity score. Patients receiving first-line gemcitabine plus cisplatin (the first-line standard of care) had a median overall survival of 8.1 months (95% CI, 7.4–8.9 months) vs 5.2 months (95% CI, 4.3–6.5 months) for first-line gemcitabine monotherapy and 8.4 months (95% CI, 6.6–10.4 months) for all other first-line treatment (Figure 3A). The 6-month, 9-month, and 12-month overall survival rates for patients receiving first-line gemcitabine monotherapy were 46.1%, 29.5%, and 19.2%, respectively; for patients receiving first-line gemcitabine plus cisplatin, they were 63.9%, 44.9%, and 33.9%, respectively. All other first-line treatment had 6-month, 9-month, and 12-month overall survival rates of 60.2%, 49.0%, and 35.1%, respectively. In addition, 54.7% of patients received gemcitabine plus cisplatin as first-line treatment, and 23.9% received gemcitabine monotherapy; median treatment duration was 3.7 months and 2.1 months, respectively. A small number of patients received capecitabine either as monotherapy or in combination with gemcitabine or platinum.

Patients receiving second-line gemcitabine monotherapy had a median overall survival of 9.7 months (95% CI, 4.2–15.5 months) vs 5.0 months (95% CI, 4.0–6.1 months) for second-line fluoropyrimidine monotherapy and 6.7 months (95% CI, 5.2–7.6 months) for all other second-line treatment (Figure 3B). The 6-month, 9-month, and 12-month overall survival rates for patients receiving second-line gemcitabine monotherapy were 60.0%, 51.3%, and 36.2%, respectively; for patients receiving second-line fluoropyrimidine monotherapy, they were 38.4%, 25.0%, and 21.4%, respectively. All other second-line treatment, including fluoropyrimidine-based or gemcitabine-based combination regimens, had 6-month, 9-month, and 12-month overall survival rates of 40.3%, 27.2%, and 25.2%, respectively. Moreover, 39.4% of patients received fluoropyrimidine-based chemotherapy as second-line treatment, and 12.3% received gemcitabine monotherapy; median treatment duration was 0.7 months and 2.3 months, respectively.

## Discussion

This study analyzed the patient and clinical characteristics, treatment patterns, and overall survival of patients diagnosed with locally advanced or metastatic BTC from January 2010 to December 2015 using SEER Medicare data. Results from this retrospective, observational cohort showed that less than half of US patients initiated systemic therapy within 90 days of diagnosis. These findings were consistent with previously reported SEER data that found that in patients with advanced gallbladder cancer, more than a third of patients received neither surgery nor chemotherapy.^16^ The low overall survival of BTC, even with treatment, may contribute to patients choosing not to initiate systemic therapy. Other possible factors contributing to the high proportion of patients electing not to initiate systemic therapy are the advanced age and stage at which patients are typically diagnosed with BTC. The most common event following first-line systemic treatment was death. Gemcitabine with or without cisplatin-based chemotherapy and fluoropyrimidine-based chemotherapy were the most common first-line and second-line systemic treatments, respectively, although second-line treatments were more varied overall. While the median overall survival was numerically higher for patients receiving “all other first-line treatment” vs gemcitabine plus cisplatin, this was likely related to selection bias.

In the absence of clear recommendations for treatment of BTC after progression on first-line therapy, information on second-line therapy in the real-world setting is sparse.^12^ The BTC treatment landscape has been evolving, with many trials initiated after this study’s cutoff date focusing on molecular profiling and biomarker-driven therapies,^19^, ^20^, ^21^ but a significant unmet need remains for patients with locally advanced or metastatic BTC for which standard chemotherapy has failed. While molecular profiling is becoming more prevalent, it was rare during this study’s observational period (2010–2015).

The US Food and Drug Administration has approved several treatments for specific genomic mutations following the time period reported in this study, including pembrolizumab in tumor mutational burden–high, microsatellite instability–high, or mismatch repair–deficient solid tumors;^19^^,^^20^ pemigatinib and infigratinib in previously treated, unresectable locally advanced, or metastatic cholangiocarcinoma harboring *FGFR2* fusions or rearrangements;^21^, ^22^, ^23^ and ivosidenib in previously treated, locally advanced, or metastatic cholangiocarcinoma with an isocitrate dehydrogenase-1 mutation.^24^ Entrectinib and larotrectinib both received US Food and Drug Administration approval for the treatment of solid tumors with an NTRK gene fusion.^25^^,^^26^ Although only a small fraction of patients are eligible for these treatments,^20^^,^^21^^,^^27^ these approvals represent a recent shift in the BTC clinical trial landscape to more targeted therapies.

For most patients with locally advanced/metastatic BTC, chemotherapy is the primary option, and recent studies have demonstrated efficacy of chemotherapy in the second-line and later setting; however, there remains no consensus on which regimen is optimal in this setting, particularly given the toxicity concerns with chemotherapy.^13^^,^^28^^,^^29^ The phase 3 ABC-06 trial (NCT01926236) showed that modified FOLFOX as a second-line treatment option may provide some benefit, although outcomes remain modest (median overall survival, 6.2 months; median progression-free survival, 4.0 months; ORR 5%) and rates of grade 3/4 treatment-related adverse events are high with chemotherapy.^13^ The phase 2b NIFTY study demonstrated efficacy improvements of 5-fluorouracil/nanoliposomal irinotecan vs 5-fluorouracil/leucovorin.^28^ In addition, a real-world study of 142 patients from a single institution in Germany demonstrated that FOLFIRINOX provided a survival benefit in patients who received it in the second-line and later setting, although only a small fraction of patients received FOLFIRINOX.^29^

A potential limitation of this study is that patients who progressed from an earlier stage of disease were excluded because in the SEER registry no details are noted about cancer progression.^16^ Another limitation is that patients in this study were all of advanced age (≥ 66 years), and while the average age of patients with BTC is > 70 years old, some patients are younger than the population in this analysis.^30^ The patient population of this cohort is demographically similar to the patients residing in the SEER coverage areas^31^ and the patient populations for other SEER studies in different solid tumor types.^32^ Using the SEER data, it is impossible to determine which patients proceeded to clinical trials following a BTC diagnosis; however, between 2010 and 2015, few clinical trials in BTC were initiated, so this patient population is likely small compared with the overall study population. Finally, while there were differences in survival among several treatment groups, all such differences in these analyses, even if statistically significant, should be interpreted as being hypothesis generating rather than causal associations. Future research may be able to provide an additional insight into specific treatment comparisons.

## Conclusion

Among newly diagnosed US patients with BTC, most did not initiate chemotherapy within 90 days of diagnosis. Overall survival from diagnosis was poor; after a mean follow-up of 8 months, 93% of the patient population had died. The variation in second-line therapies reflects the lack of consensus on how to treat older patients with advanced BTC that progressed after first-line therapy. Additional studies with more recent data will need to be conducted to evaluate the effect of targeted agents/biomarker profiling on the treatment patterns of patients with locally advanced or metastatic BTC.
