# Supplementary material for: Treatment Patterns and Survival in Locally Advanced or Metastatic Biliary Tract Cancer Using SEER Medicare Data
Source: Gastro Hep Adv. 2023 Jan 21;2(4):580–7. doi: 10.1016/j.gastha.2023.01.009 (PMC11307571; doi:10.1016/j.gastha.2023.01.009)
Supplement: Supplementary File [file mmc1.pdf]

Supplementary Table 1. Cox Proportional Hazards Model for Biliary Tract Cancer at Diagnosis

Variable	Estimate (hazard ratio)	Standard error	Lower CI	Upper CI	P value
Age (per year)	1.036	0.004	1.029	1.043	<0.001
Male (vs female)	1.129	0.048	1.028	1.239	0.011
Black (vs White)	1.076	0.081	0.917	1.262	0.369
Asian (vs White)	0.943	0.085	0.799	1.112	0.484
All other (vs White)	0.842	0.222	0.546	1.301	0.439
Any mobility limitation (vs none)	1.212	0.059	1.080	1.361	0.001
>30% Below poverty in census tract (vs ≤30%)	1.263	0.083	1.074	1.485	0.005
NCI comorbidity score (per unit, range 0-9)	1.055	0.011	1.031	1.079	<0.001
History of cancer (vs none)	0.757	0.092	0.632	0.907	0.003
Intrahepatic (vs extrahepatic)	0.804	0.061	0.712	0.906	<0.001
Gallbladder (vs extrahepatic)	1.117	0.056	1.001	1.247	0.048
Stage 4 (vs stage 3)	1.174	0.082	1.001	1.378	0.049

Supplementary Figure 1. Study flow diagram. BTC, biliary tract cancer; HMO, health maintenance organization; SEER, Survival, Epidemiology, and End Results Program.

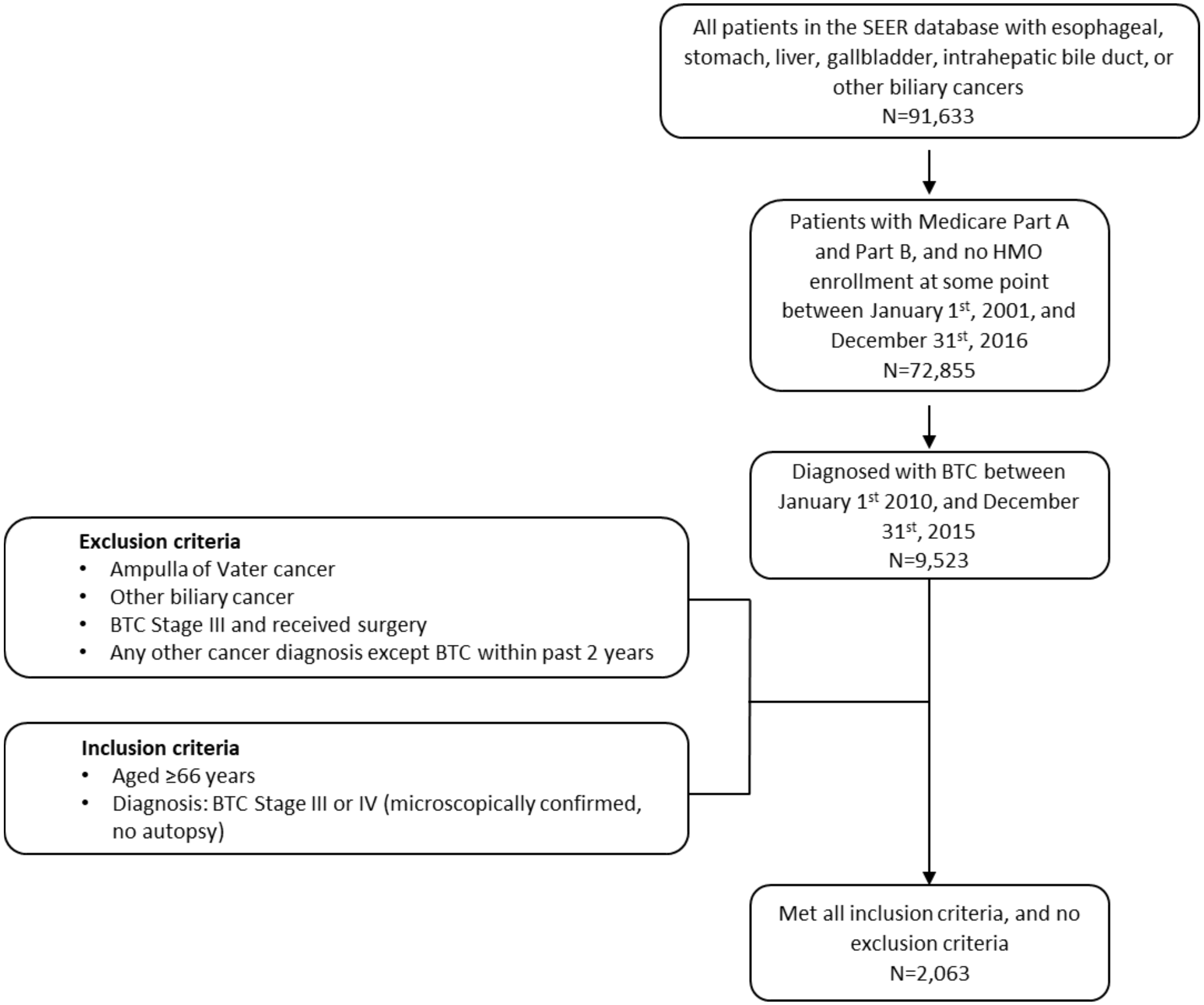

Supplementary Figure 2. Time to first event after diagnosis (A) and after end of first-line therapy (B) for patients with a National Cancer Institute comorbidity index <2.

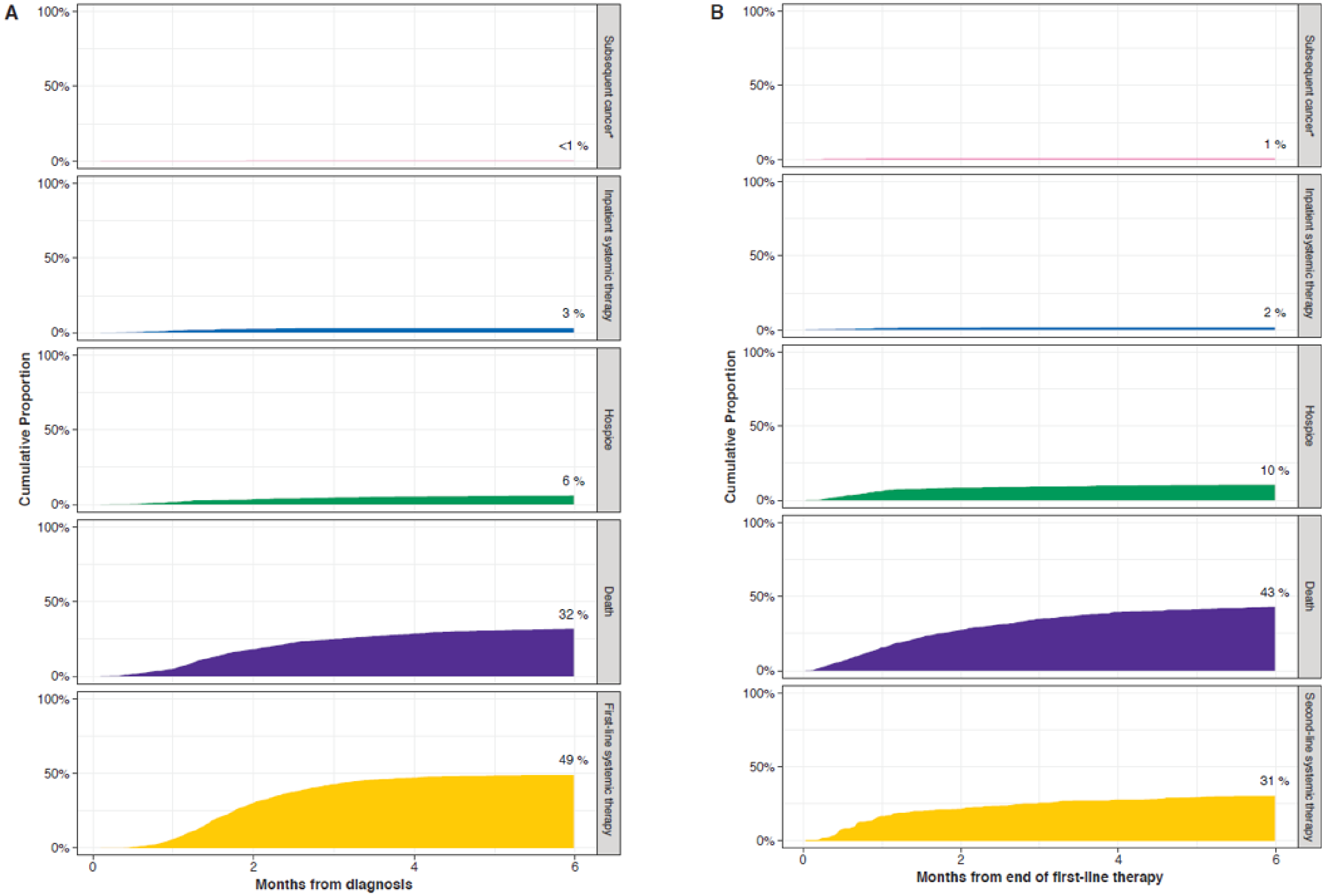

*Subsequent cancer does not refer to metastasis, only an additional cancer diagnosis.
